# Supplementary material for: Integrin CD103 reveals a distinct developmental pathway of autoreactive thymocytes in TCR transgenic mice
Source: Nat Commun. 2025 Jul 18;16:6627. doi: 10.1038/s41467-025-61922-8 (PMC12274481; doi:10.1038/s41467-025-61922-8)
Supplement: Supplementary file 1 — Supplementary Information [file 41467_2025_61922_MOESM1_ESM.pdf]

Supplementary figures

Integrin CD103 reveals a distinct developmental pathway of autoreactive thymocytes in TCR transgenic mice

Nurcin Liman[1], Can Li[1], Megan A. Luckey[1], Hilary R. Keller[1], Jie Li[1], William Hajjar[1], Jan Wisniewski[1], Michael Kruhlak[2], Vanja Lazarevic[1], and Jung-Hyun Park[1,*]

[1] Experimental Immunology Branch, Center for Cancer Research, National Cancer Institute, NIH, Bethesda, MD 20892; [2] Laboratory of Cancer Biology and Genetics, Center for Cancer Research, National Cancer Institute, NIH, Bethesda, MD 20892

Supplementary Figure 1

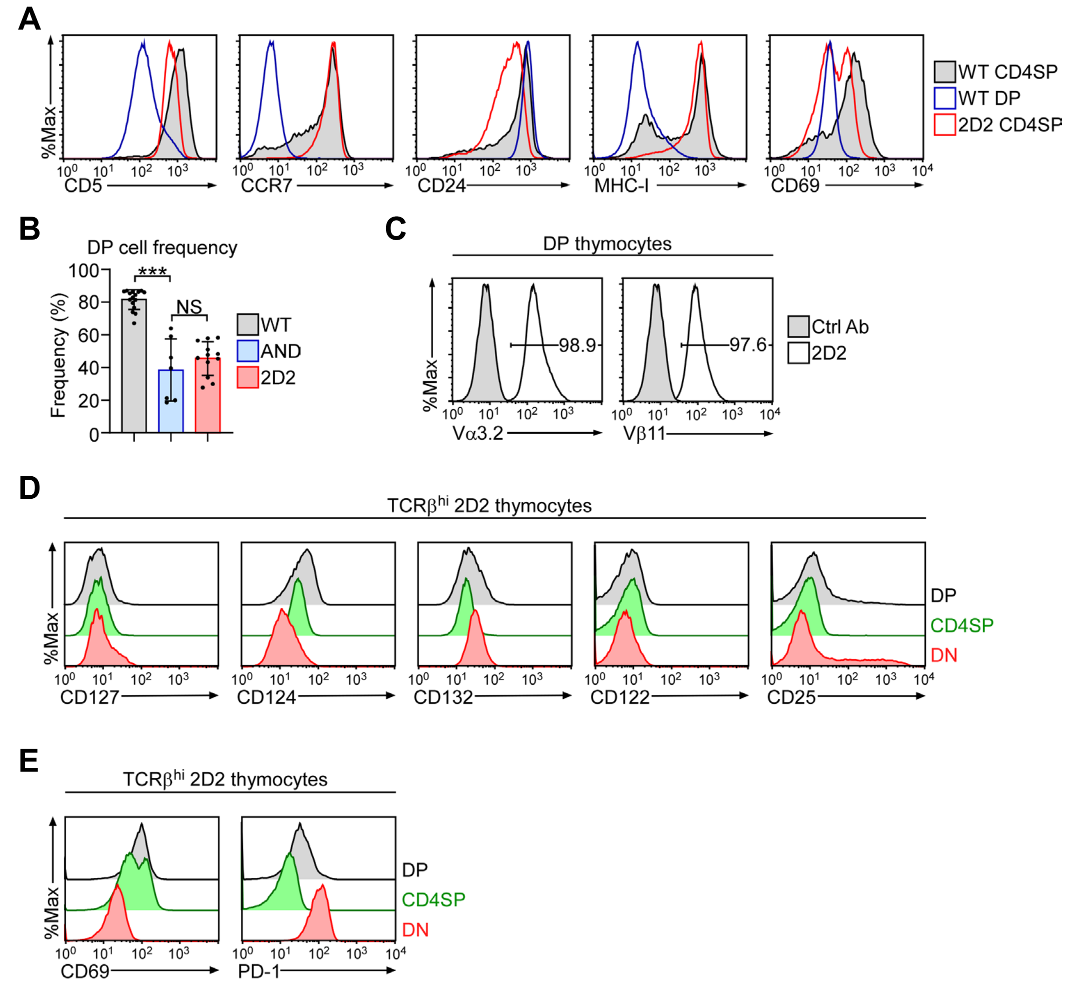

Suppl. Fig. 1. Thymocyte development in 2D2 TCR transgenic mice
A. Histograms show the cell surface expression of CD5, CCR7, CD24, MHC-I ($H\text{-}2K^bD^b$), and CD69 on CD4SP thymocytes of 2D2 and WT mice compared to that of DP thymocytes of WT mice. Results are representative of 4 independent experiments.
B. Bar graphs show the frequency of DP thymocytes in WT (n=16), AND (n=7), and 2D2 (n=12) mice as a summary of 16 independent experiments. Data are presented as mean values ± Standard Deviation (SD). ***$P < 0.001$; NS, not significant.
C. Histograms show the clonotypic TCRα Vα3.2 (left) and TCRβ Vβ11 (right) expression on 2D2 DP thymocytes. Results are representative of 3 independent experiments. Ctrl Ab, control antibody.
D, E. Histograms show the cell surface expression of the cytokine receptors CD127, CD124, CD132, CD122, and CD25 (**D**), and the activation markers CD69 and PD-1 (**E**) on TCRβ^{hi}–gated 2D2 TCR transgenic thymocytes that are either DP, CD4SP and DN for CD4 and CD8 coreceptor expression. Results are representative of 2 independent experiments.

Supplementary Figure 2

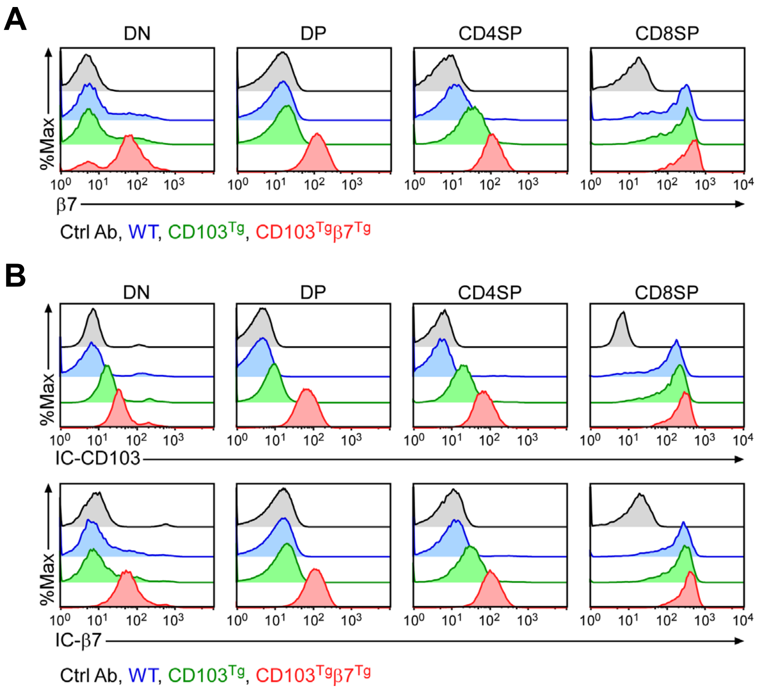

Suppl. Fig. 2. Surface and intracellular staining of CD103, β7 expression in thymocyte subsets
A. Histograms show surface expression of integrin β7 on DN, DP, CD4SP, and CD8SP thymocytes of WT, $CD103^{Tg}$, and $CD103^{Tg}β7^{Tg}$ mice. Results are representative of 6 independent experiments. Ctrl Ab, control antibody.
B. Histograms show the intracellular (IC) expression of CD103 (IC-CD103) and β7 (IC-β7) in DN, DP, CD4SP, and CD8SP thymocytes of WT, $CD103^{Tg}$, and $CD103^{Tg}β7^{Tg}$ mice. Results are representative of 2 independent experiments. Ctrl Ab, control antibody.

Supplementary Figure 3

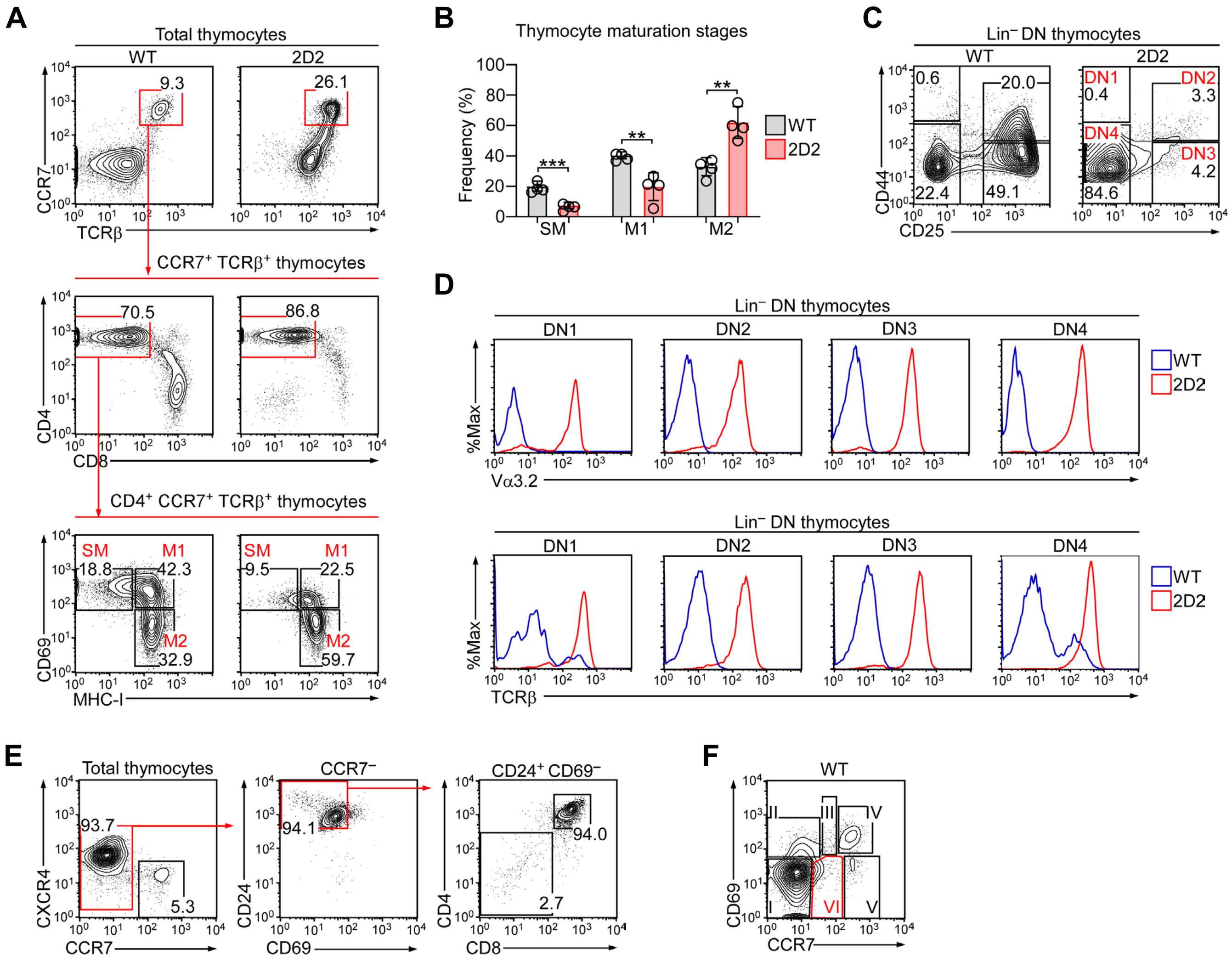

Suppl. Fig. 3. Pre- and post-selection development of 2D2 TCR transgenic thymocytes

A. Total thymocytes of 2D2 and WT mice were assessed for CCR7 versus TCRβ expression (top), among which post-selection CCR7$^+$TCRβ$^+$ cells were plotted for CD4 versus CD8 expression (middle). Post-selection CD4SP cells were then examined for CD69 versus MHC-I (H-2K^{b}D^b) expression to identify semi-mature (SM; CD69$^+$ MHC-I-negative), stage 1 mature (M1; CD69$^+$ MHC-I$^+$), and stage 2 mature (M2; CD69-negative MHC-I$^+$) CD4SP thymocytes (bottom). Results are representative of 3 independent experiments.

B. Bar graph shows the frequencies of SM, M1, and M2 CD4SP cells among post-selection CCR7$^+$TCRβ$^+$ thymocytes of WT (n=4) and 2D2 (n=4) mice. Results are a summary of 3 independent experiments. Data are presented as mean values ± SD. ***P < 0.001; **P < 0.01.

C. The contour plots show the CD44 versus CD25 profiles of lineage marker negative (Lin$^-$) DN thymocytes of WT and 2D2 mice. DN1 subsets were identified as DN1 (CD44$^+$CD25$^-$), DN2 (CD44$^+$CD25$^+$), DN3 (CD44$^-$CD25$^+$), and DN4 (CD44$^-$CD25$^-$). Results are representative of 2 independent experiments.

D. The histograms show clonotypic TCRα Vα3.2 (top) and TCRβ (bottom) expression on DN1 to DN4 subsets of WT (blue) and 2D2 (red) thymocytes. Results are representative of 2 independent experiments.

E. The contour plots show the serial gating strategy to identify pre-selection DP thymocytes. Total thymocytes were first plotted for CXCR4 versus CCR7 expression to identify pre-selection (*i.e.*, CXCR4$^+$CCR7$^-$) thymocytes (left), which were re-gated for the lack of CD69 but high levels of CD24 (middle) and finally assessed for CD4 versus CD8 expression to gate on DP thymocytes (right).

F. The contour plot shows the CD69 versus CCR7 profile of total thymocytes in WT mice. The thymocyte developmental stages are identified as I (CD69$^-$CCR7$^-$), II (CD69$^+$CCR7$^-$), III (CD69$^+$CCR7^{int}), IV (CD69$^+$CCR7$^+$), and V (CD69$^+$CCR7$^+$) from least mature to most mature, as described previously. We identified a new population, Population VI (CD69$^-$CCR7^{int}, marked in red), that mostly correspond to negatively selected cells.

Supplementary Figure 4

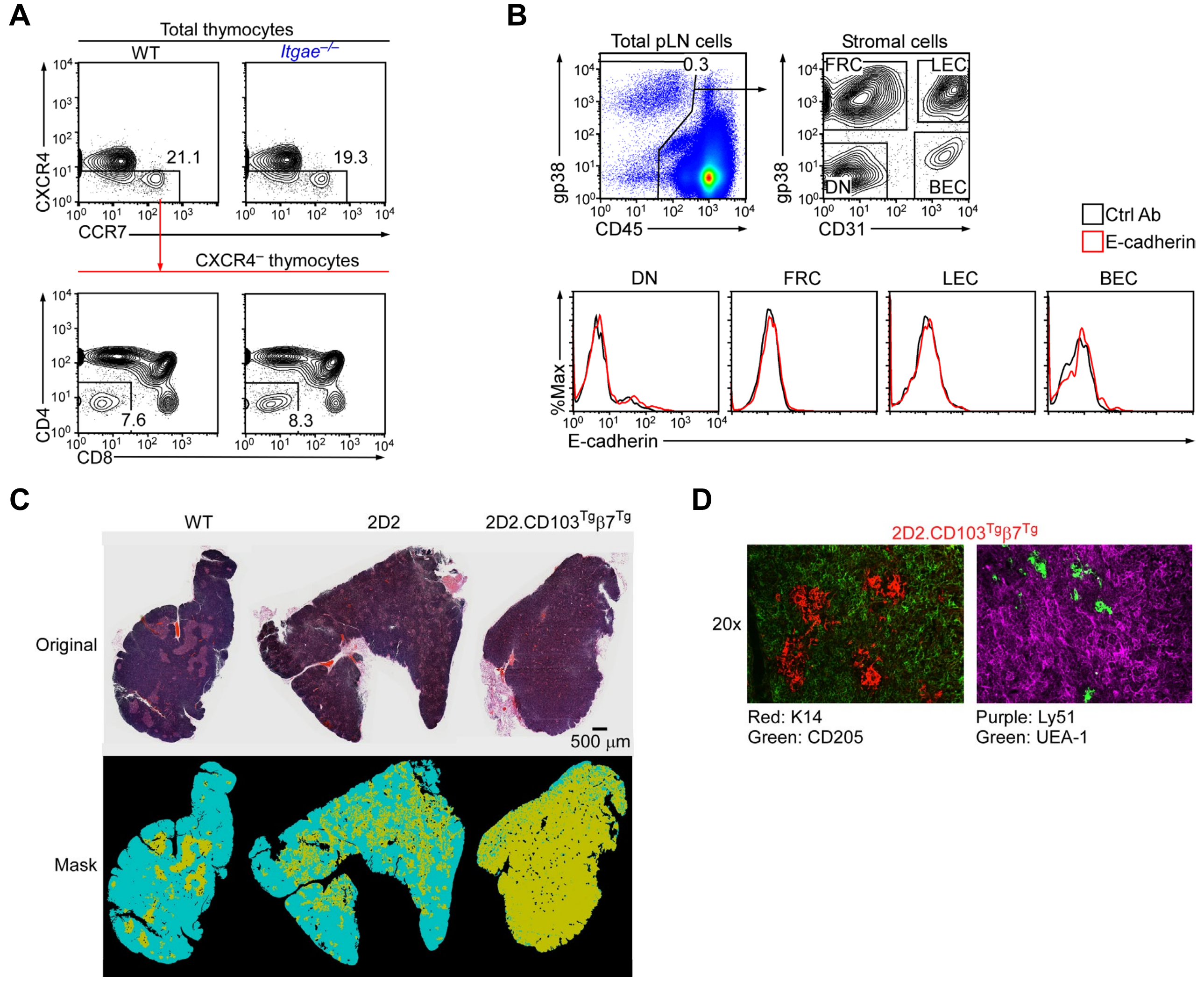

Suppl. Fig. 4. Histochemistry and immunohistochemistry analyses of thymic section from WT, 2D2, and 2D2.CD103^{Tg}β7^{Tg} mice

A. The contour plots show the gating strategy to identify post-selection DN thymocytes in WT and *Itgae*$^{-/-}$ mice. Total thymocytes were first plotted for CXCR4 versus CCR7 expression to identify post-selection (*i.e.*, CXCR4^{-}) thymocytes (top), which were then plotted for CD4 versus CD8 expression to identify mature DN thymocytes (bottom).

B. E-cadherin expression was assessed on different subsets of CD45-negative stromal cells from peripheral LNs of WT mice. FRC, LEC, and BEC stromal cells were identified based on their gp38, CD31 expression as indicated. FRCs, fibroblastic reticular cells; LECs, lymphatic endothelial cells; BECs, blood endothelial cells. The data are representative of 3 independent experiments.

C. Hematoxylin and eosin staining of thymic sections of the indicated mice (top, Original) and digitized mosaic images identifying the medulla and cortex regions by quantifying pixel densities using the "Histological_Mosaic_Assembly_and_Annotation" ImageJ macro (bottom, Mask). Areas of thymus identified as medulla by the macro function are shown in yellow and those of the cortex are shown in cyan.

D. Immunohistochemistry shows thymic sections of 2D2.CD103^{Tg}β7^{Tg} mice stained for anti-K14 (red) and anti-CD205 (green) that identify the medullary and cortical areas, respectively (left). The same thymic sections were also stained with anti-Ly51 (purple) and anti-UEA-1 (green) to map the medullary and cortical areas, respectively (right).

Supplementary Figure 5

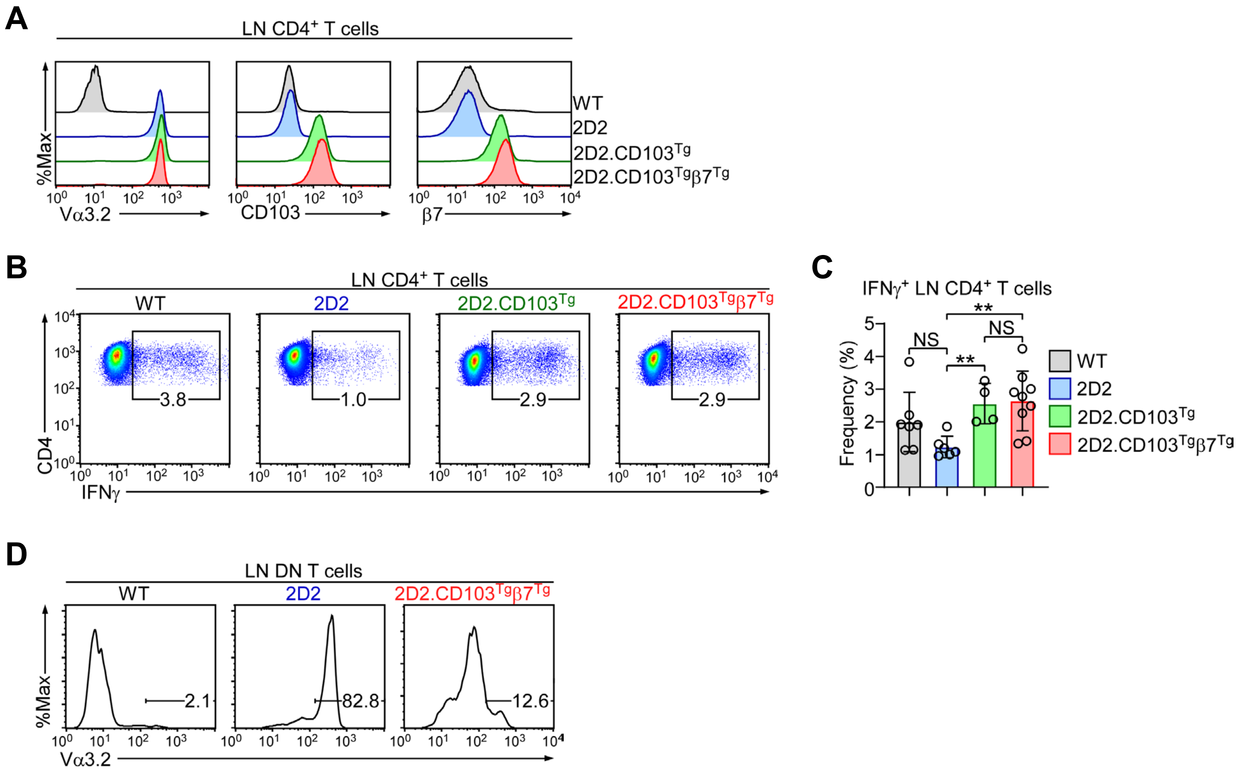

Suppl. Fig. 5. Phenotypic and functional characterization of 2D2.CD103^{Tg}β7^{Tg} CD4 LN T cells
A. Histograms show the expression of 2D2 clonotypic TCRα (Vα3.2), CD103, and β7 on LN CD4 T cells of WT, 2D2, 2D2.CD103^{Tg}, and 2D2.CD103^{Tg}β7^{Tg} mice. Results are representative of 3 independent experiments.
B, C. Dot plots are representative (**B**), and bar graph shows the summary (**C**) of intracellular IFN-γ expression in PMA + ionomycin-stimulated LN CD4 T cells of WT (n=7), 2D2 (n=6), 2D2.CD103^{Tg} (n=4), and 2D2.CD103^{Tg}β7^{Tg} (n=9) mice. Results are representative of 4 independent experiments. Data are presented as mean values ± SD. **, *P* < 0.01; NS, not significant.
D. The histograms show the expression of 2D2 clonotypic TCRα (Vα3.2) on LN DN T cells of WT, 2D2, and 2D2.CD103^{Tg}β7^{Tg} mice. Results are representative of 3 independent experiments.

Supplementary Figure 6

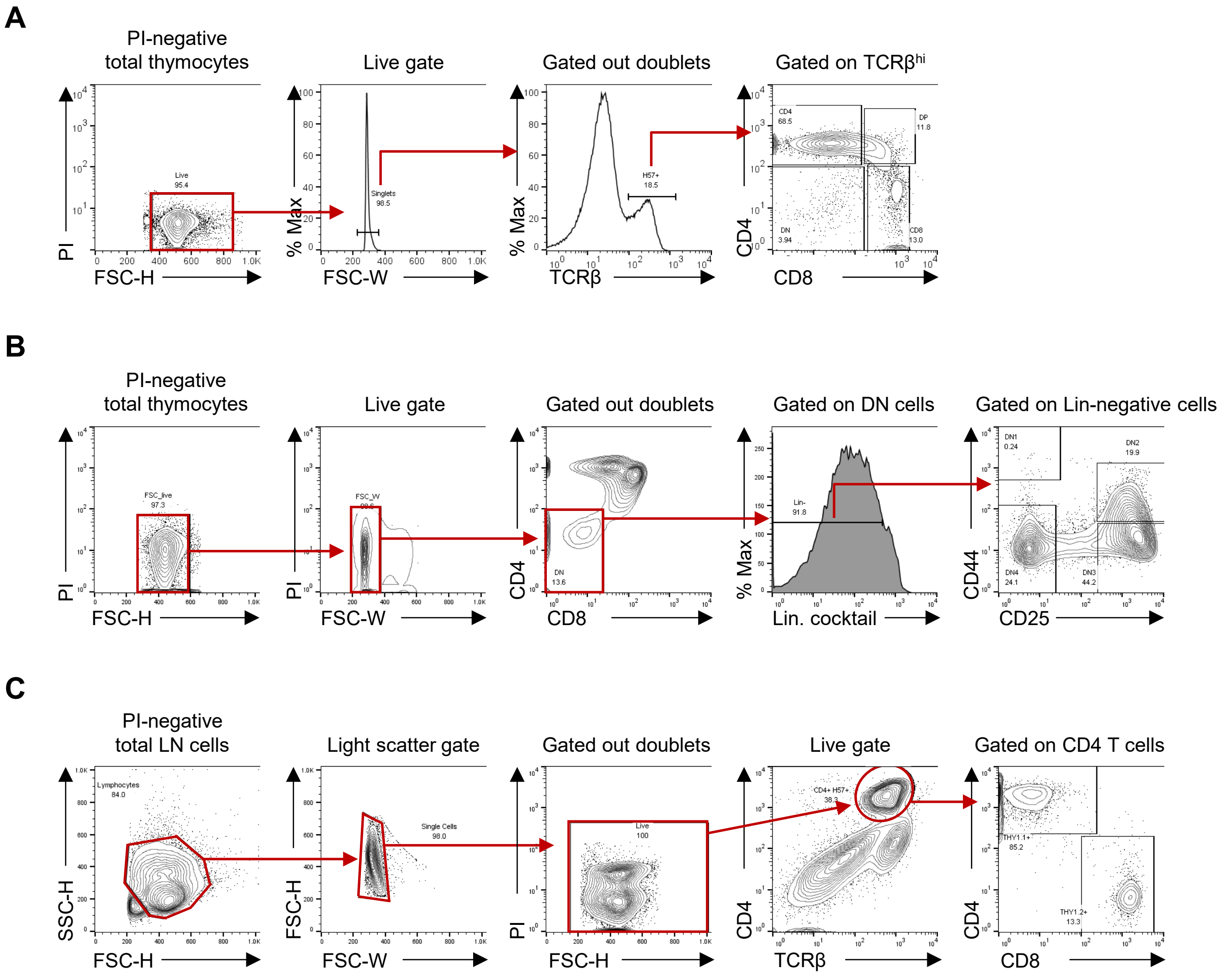

Suppl. Fig. 6. Gating strategies used for identifying cell populations
A. Gating strategy to identify TCRβ^{hi} thymocytes for downstream analysis of surface markers as presented in Figures 1F, 1G, 1J, 1I, and Suppl. Fig. 1D and 1E.
B. Gating strategy to identify lineage marker-negative DN thymocyte subsets as presented in Suppl. Fig. 3C.
C. Gating strategy to identify donor CD4 T cells in *Rag2*-deficient host mice as presented in Figure 7F.
