# Supplementary material for: Integrin CD103 reveals a distinct developmental pathway of autoreactive thymocytes in TCR transgenic mice
Source: Nat Commun. 2025 Jul 18;16:6627. doi: 10.1038/s41467-025-61922-8 (PMC12274481; doi:10.1038/s41467-025-61922-8)
Supplement: Supplementary file 2 — Description of Additional Supplementary Files [file 41467_2025_61922_MOESM2_ESM.pdf]

Description of Additional Supplementary Files

Supplementary Data 1: Antibody dilution chart
