# Supplementary material for: Integrin CD103 reveals a distinct developmental pathway of autoreactive thymocytes in TCR transgenic mice
Source: Nat Commun. 2025 Jul 18;16:6627. doi: 10.1038/s41467-025-61922-8 (PMC12274481; doi:10.1038/s41467-025-61922-8)
Supplement: Supplementary file 4 — Reporting Summary [file 41467_2025_61922_MOESM4_ESM.pdf]

nature portfolio

Corresponding author(s): Jung-Hyun Park

Last updated by author(s): Jun 13, 2025

Reporting Summary

Nature Portfolio wishes to improve the reproducibility of the work that we publish. This form provides structure for consistency and transparency in reporting. For further information on Nature Portfolio policies, see our Editorial Policies and the Editorial Policy Checklist.

Statistics

For all statistical analyses, confirm that the following items are present in the figure legend, table legend, main text, or Methods section.

n/a	Confirmed	
☐	☒	The exact sample size (n) for each experimental group/condition, given as a discrete number and unit of measurement
☐	☒	A statement on whether measurements were taken from distinct samples or whether the same sample was measured repeatedly
☐	☒	The statistical test(s) used AND whether they are one- or two-sided<br>*Only common tests should be described solely by name; describe more complex techniques in the Methods section.*
☒	☐	A description of all covariates tested
☒	☐	A description of any assumptions or corrections, such as tests of normality and adjustment for multiple comparisons
☐	☒	A full description of the statistical parameters including central tendency (e.g. means) or other basic estimates (e.g. regression coefficient) AND variation (e.g. standard deviation) or associated estimates of uncertainty (e.g. confidence intervals)
☐	☒	For null hypothesis testing, the test statistic (e.g. F, t, r) with confidence intervals, effect sizes, degrees of freedom and P value noted<br>*Give P values as exact values whenever suitable.*
☒	☐	For Bayesian analysis, information on the choice of priors and Markov chain Monte Carlo settings
☒	☐	For hierarchical and complex designs, identification of the appropriate level for tests and full reporting of outcomes
☒	☐	Estimates of effect sizes (e.g. Cohen's d, Pearson's r), indicating how they were calculated

Our web collection on statistics for biologists contains articles on many of the points above.

Software and code

Policy information about availability of computer code

Data collection	BD LSRFortessa and BD LSRII flow cytometers were used to collect flow cytometry samples. Microscopy data was acquired on a Zeiss Axio Observer microscope.
Data analysis	Flow cytometry data was analyzed using FlowJo (version 10.10.0) and statistical analyses were done with GraphPad Prism (version 10). Histology images were analyzed using Zen Microscopy Software (Zeiss) and Arivis Pro (Zeiss) or by ImageJ macro (NIH).

For manuscripts utilizing custom algorithms or software that are central to the research but not yet described in published literature, software must be made available to editors and reviewers. We strongly encourage code deposition in a community repository (e.g. GitHub). See the Nature Portfolio guidelines for submitting code & software for further information.

Data

Policy information about availability of data

All manuscripts must include a data availability statement. This statement should provide the following information, where applicable:

- Accession codes, unique identifiers, or web links for publicly available datasets
- A description of any restrictions on data availability
- For clinical datasets or third party data, please ensure that the statement adheres to our policy

Data will be made available on request, as indicated in the 'Data availability' section of the manuscript.

Research involving human participants, their data, or biological material

Policy information about studies with human participants or human data. See also policy information about sex, gender (identity/presentation), and sexual orientation and race, ethnicity and racism.

Reporting on sex and gender	n/a
Reporting on race, ethnicity, or other socially relevant groupings	n/a
Population characteristics	n/a
Recruitment	n/a
Ethics oversight	n/a

Note that full information on the approval of the study protocol must also be provided in the manuscript.

Field-specific reporting

Please select the one below that is the best fit for your research. If you are not sure, read the appropriate sections before making your selection.

☒ Life sciences ☐ Behavioural & social sciences ☐ Ecological, evolutionary & environmental sciences

For a reference copy of the document with all sections, see nature.com/documents/nr-reporting-summary-flat.pdf

Life sciences study design

All studies must disclose on these points even when the disclosure is negative.

Sample size	Sample sizes were estimated based on our previous studies. Whever possible, at least 3-4 mice per group were used in each experiment. Sample sizes are described in the figure legends.
Data exclusions	No data were excluded.
Replication	Reproducibility were ensured with at least two independent experiments. Repeated numbers of experiments were described in the figure legends.
Randomization	All animals in different groups were used randomly based on their genotype.
Blinding	No blinding was performed, which allowed appropriate grouping of control and genetically modified mice. This also ensured appropriate sample sizes with the minimum number of mice required, in accordance with ethical issues.

Reporting for specific materials, systems and methods

We require information from authors about some types of materials, experimental systems and methods used in many studies. Here, indicate whether each material, system or method listed is relevant to your study. If you are not sure if a list item applies to your research, read the appropriate section before selecting a response.

Materials & experimental systems

n/a	Involved in the study	
☐	☒	Antibodies
☒	☐	Eukaryotic cell lines
☒	☐	Palaeontology and archaeology
☐	☒	Animals and other organisms
☒	☐	Clinical data
☒	☐	Dual use research of concern
☒	☐	Plants

Methods

n/a	Involved in the study	
☒	☐	ChIP-seq
☐	☒	Flow cytometry
☒	☐	MRI-based neuroimaging

Antibodies

Antibodies used	1. Anti-mouse TCRβ chain antibody (clone: H57-597) Alexa Fluor® 594, Biolegend, Cat#:109238<br>2. Anti-mouse TCRβ chain antibody (clone: H57-597) BD OptiBuild™ BV786,BD, Cat#: 742484

3. Anti-mouse TCRβ chain antibody (clone: H57-597) Brilliant Violet 421™, Biolegend, Cat#: 109230
4. Anti-mouse CD4 (clone: GK1.5) PE-Cyanine7, Tonbo, Cat#: 60-0041-U100
5. Anti-mouse CD4 (clone: RM4-5) BD Horizon™ BV786, BD, Cat#: 563727
6. Anti-mouse CD8α antibody (clone: 53-6.7), APC-eFluor™ 780, eBioscience™ Invitrogen, Cat#: 47-0081-82
7. Anti-mouse CD8α antibody (clone: 53-6.7) Brilliant Violet 785™, Biolegend, Cat#: 100750
8. Anti-mouse CD24 antibody (clone: M1/69) Alexa Fluor® 594, Biolegend, Cat#:101834
9. Anti-mouse CD24 antibody (clone: M1/69) eFluor™ 450, eBioscience™ Invitrogen, Cat#: 48-0242-82
10. Anti-mouse CD24 antibody, (clone: M1/69) PE/Cyanine7, Biolegend, Cat#: 101822
11. Anti-mouse CD69 antibody (clone: H1.2F3) APC, Biolegend, Cat#: 104514
12. Anti-mouse CD69 antibody (clone: H1.2F3) PE, eBioscience™ Invitrogen, Cat#: 12-0691-82
13. Anti-mouse TCR Vα3.2 [b, c] antibody (clone: RR3-16) FITC, Biolegend, Cat#: 135404
14. Anti-mouse TCR Vβ11 antibody (clone: RR3-15) FITC, BD, Cat#: 553197
15. Anti-mouse CD103 (Integrin alpha E) antibody (clone: 2E7), PE, eBioscience™ Invitrogen, Cat#: 12-1031-83
16. Anti-mouse CD103 (Integrin alpha E) antibody (clone: 2E7), FITC, eBioscience™, Invitrogen, Cat#: 11-1031-85
17. Anti-mouse CD49d antibody (clone: R1-2) FITC, Biolegend, Cat#:103606
18. Anti-mouse CD49d (clone: 9C10) PE, BD, Cat#: 557420
19. Anti-mouse CD103 (Integrin alpha E) antibody (clone: 2E7) Alexa Fluor® 647, Biolegend, Cat#:121410
20. Anti-human/mouse integrin β7 antibody (clone: FIB504), PE, eBioscience™ Invitrogen, Cat#: 12-5867-42
21. Anti-human/mouse integrin β7 antibody (clone: FIB504) APC, Biolegend, Cat#: 321208
22. Anti-mouse CD29 (Integrin beta 1) antibody (eBioHMb1-1 (clone: HMb1-1)) APC, eBioscience™ Invitrogen, Cat#: 17-0291-82
23. Anti-mouse CD184 (CXCR4) antibody (clone: 2B11) PE, eBioscience™ Invitrogen, Cat#: 12-9991-82
24. Anti-mouse CD197 (CCR7) antibody (clone: 4B12) biotin, eBioscience™, Invitrogen, Cat#: 13-1971-82
25. Anti-mouse/human CD44 antibody (clone: IM7) Brilliant Violet 421™, Biolegend, Cat#: 103040
26. Anti-mouse/human CD44 antibody (clone: IM7) APC, Biolegend, Cat#: 103012
27. Anti-mouse CD62L antibody (clone: MEL-14) APC, Biolegend, Cat#: 104412
28. Anti-mouse CD279 (PD-1) antibody (clone: 29F.1A12) PE, Biolegend, Cat#: 135206
29. Anti-mouse CD5 antibody (clone: 53-7.3) FITC, eBioscience™ Invitrogen, Cat#: 11-0051-82
30. Anti-mouse CD5 antibody (clone: 53-7.3) APC, eBioscience™ Invitrogen, Cat#: 17-0051-82
31. Anti-mouse H-2Kb (clone: AF6-88.5) PE, BD, Cat#: 553570
32. Anti-mouse IFN gamma antibody (clone: XMG1.2) PE-Cyanine7, eBioscience™ Invitrogen, Cat#: 25-7311-41
33. Anti-mouse IFN gamma antibody (clone: XMG1.2) FITC, Tonbo, Cat#: 35-7311-U100
34. Anti-mouse RORγt antibody (clone: Q31-378) BD Horizon™ BV786, BD, Cat#: 564723
35. Anti-mouse CD16/CD32 antibody (Mouse BD Fc Block™) purified (clone: 2.4G2), BD, Cat#: 553142
36. Anti-mouse CD8α antibody (clone: 53-6.7) biotin, BD, Cat#: 553029
37. Anti-mouse CD45R/B220 antibody (clone: RA3-6B2) biotin, BD, Cat#: 553086
38. Anti-mouse NK1.1 antibody (clone: 553165) PE, BD, Cat#: 553165
39. Anti-mouse CD11b antibody (clone: M1/70) FITC, eBioscience™ Invitrogen, Cat#: 14-0112-82
40. Anti-mouse Ly-6G/Ly-6C antibody (clone: RB6-8C5), Biotin, eBioscience™ Invitrogen, Cat#: 13-5931-82
41. Anti-mouse γδ TCR antibody (clone: GL3) biotin, BD, Cat#: 553176
42. Anti-mouse CD45R antibody (clone: RA3-6B2) Pacific Blue™, BD, Cat#: 558108
43. Anti-mouse CD25 antibody (clone: PC61.5) PE-Cyanine7, eBioscience™ Invitrogen, Cat#: 25-0251-82
44. Anti-mouse CD45 antibody (clone: 30-F11) APC-eFluor™ 780, eBioscience™ Invitrogen, Cat#: 47-0451-82
45. Anti-mouse CD326 (EpCAM) antibody (clone: G8.8) eFluor™ 450, eBioscience™ Invitrogen, Cat#: 48-5791-82
46. Anti-mouse/human Helios antibody (clone: 22F6) FITC, BioLegend, Cat#: 137204
47. Anti-mouse NK1.1 antibody (clone: PK136) PE-Cyanine7, eBioscience™ Invitrogen, Cat#: 25-5941-82
48. Anti-mouse γδ TCR antibody (clone: GL3) FITC, BD, Cat#: 553177
49. Anti-mouse CD127 antibody (clone: A7R34) FITC, eBioscience™ Invitrogen, Cat#: 11-1271-85
50. Anti-mouse CD124 antibody (clone: mIL4R-M1) PE, BD, Cat#: 552509
51. Anti-mouse CD132 antibody (clone: 4G3) PE, BD, Cat#: 554457
52. Anti-mouse CD122 (IL-2Rβ) antibody (clone: TM-β1) FITC, BioLegend, Cat#: 123208
53. Anti-rat CD90/mouse CD90.1 antibody (clone: OX7) FITC, BD, Cat#: 561973
54. Anti-mouse CD90.2 (Thy-1.2) antibody (clone: 53-2.1) APC-eFluor™ 780, eBioscience™ Invitrogen, Cat#: 47-0902-82
55. Anti-mouse CD8β.2 antibody (clone: 53-5.8) Pacific Blue™, BioLegend, Cat#: 140414
56. Anti-mouse CD324 antibody (E-Cadherin) (clone: DECMA-1) PE, BD, Cat#: 567052
57. Anti-mouse podoplanin antibody (clone: 8.1.1) PE, eBioscience™ Invitrogen, Cat#: 12-5381-82
58. Anti-mouse CD31 antibody (clone: 390) Alexa Fluor™ 647, Invitrogen Cat#: A14716
59. Anti-mouse Ikaros antibody (clone: 2A9) PE, Biolegend, Cat#: 653304
60. Anti-mouse Ly-6G/Ly-6C antibody (clone: RB6-8C5) Pacific Blue™, Invitrogen, Cat#: RM3028
61. Anti-mouse CD11b antibody (clone: M1/70) PE, eBioscience™, Invitrogen, Cat#: 12-0112-82
62. CaspGLOW™ Fluorescein Active Caspase-3 Staining Kit, Invitrogen, 88-7004
63. Streptavidin, Alexa Fluor™ 594 conjugate, Invitrogen, Cat#: S11227

Validation

All the antibodies are available from commercial sources and were validated by the corresponding manufacturers. We selected antibody clones that have been used in the literature. We also titrated all antibodies prior to experiments.

Animals and other research organisms

Policy information about studies involving animals; ARRIVE guidelines recommended for reporting animal research, and Sex and Gender in Research

Laboratory animals

C57BL/6 (B6) mice were obtained from the Charles River Laboratories (Frederick, MD). OT-II TCR transgene, 2D2 TCR transgene, AND TCR transgene, Rag2–/–, and Itgae–/– mice were procured from the Jackson Laboratories (JAX#004194, #006912, #002761, #008449 and #006144, respectively). T cell-specific CD103 and β7 transgenic mice were generated in-house and had been previously reported

(doi.org/10.1007/s00018-021-03877-9). Ikzf1fl/fl mice were described previously (doi.org/10.1084/jem.20131735) and kindly provided by Drs. Chan and Kastner (INSERM, France), and were crossbred in-house with CD4Cre mice procured from the Jackson Laboratories (#022071). HY TCR transgenic mice were described previously (doi.org/10.1038/333742a0)
All mice were 4 - 12 weeks of age. All mice were cared for in accordance with NIH guidelines under SPF conditions. Animals were housed in ventilated racks with an automatic watering system and ad libitum access to food on a 12-hours light/dark cycle under ambient conditions. At the end of the experiments, mice were euthanized by CO2 inhalation.

Wild animals	No wild animals were used in this study.
Reporting on sex	Both male and female mice were used.
Field-collected samples	n/a
Ethics oversight	All mice were cared for in accordance with NIH guidelines. All animal procedures reported in this study that were performed by NCI-CCR affiliated staff were approved by the NCI Animal Care and Use Committee (ACUC) and in accordance with federal regulatory requirements and standards. All components of the intramural NIH ACU program are accredited by AAALAC International.

Note that full information on the approval of the study protocol must also be provided in the manuscript.

Plants

Seed stocks	n/a
Novel plant genotypes	n/a
Authentication	n/a

Flow Cytometry

Plots

Confirm that:

☐ The axis labels state the marker and fluorochrome used (e.g. CD4-FITC).

☒ The axis scales are clearly visible. Include numbers along axes only for bottom left plot of group (a 'group' is an analysis of identical markers).

☒ All plots are contour plots with outliers or pseudocolor plots.

☒ A numerical value for number of cells or percentage (with statistics) is provided.

Methodology

Sample preparation	For live cell analysis, dead cells were excluded by adding propidium iodide (PI) before running the samples on flow cytometers. For fixed cell staining and analysis, cells were stained with Live/Dead Ghost Dye Violet 510 (Tonbo) for exclusion of dead cells, followed by surface staining and fixation with Foxp3 fixation buffer for transcription factors (eBioscience) or intracellular fixation buffer for cytokines (eBioscience). Afterward, cells were permeabilized using the Foxp3 intracellular staining kit, following the manufacturer's instructions (eBioscience). Excess reagents were removed by extensive washing in FACS buffer (0.1% BSA, 0.1% sodium azide in HBSS) before flow cytometric analysis.
Instrument	Fluorescence antibody-stained single-cell suspensions were analyzed using LSRFortessa or LSRII flow cytometers (BD Biosciences).
Software	Flow cytometry data was analyzed using FlowJo (version 10.10.0)
Cell population abundance	n/a
Gating strategy	To identify live singlet cells, lymphocytes were gated in by FSC-H/SSC-H, singlets were gated in by FSC-H/FSC-W, and live cells were gated in by FSC-H/PI gates. All gates were defined based on Fluorescence Minus One (FMO) or secondary antibody controls. Gates in fluorescence channels were based on clear separation between negative and positive populations. The strategies are indicated in the Supplemental Figures.

☒ Tick this box to confirm that a figure exemplifying the gating strategy is provided in the Supplementary Information.
